# Green HPLC Enantioseparation of Chemopreventive Chiral Isothiocyanates Homologs on an Immobilized Chiral Stationary Phase Based on Amylose tris-[(*S*)-α-Methylbenzylcarbamate]

**DOI:** 10.3390/molecules29122895

**Published:** 2024-06-18

**Authors:** Francesca Romana Mammone, Alessia Panusa, Roberta Risoluti, Roberto Cirilli

**Affiliations:** 1 National Centre for the Control and Evaluation of Medicines, Chemical Medicines Unit, Istituto Superiore di Sanità, Viale Regina Elena 299, 00161 Rome, Italy; francescaromana.mammone@uniroma1.it (F.R.M.); alessia.panusa@iss.it (A.P.); 2 Department of Chemistry, “Sapienza” University of Rome, P.le A. Moro 5, 00185 Rome, Italy; roberta.risoluti@uniroma1.it

**Keywords:** CHIRALPAK IH-3, green enantioselective HPLC, iberin, sulforaphane, alyssin, hesperin, absolute configuration

## Abstract

Sulforaphane is a chiral phytochemical with chemopreventive properties. The presence of a stereogenic sulfur atom is responsible for the chirality of the natural isothiocyanate. The key role of sulfur chirality in biological activity is underscored by studies of the efficacy of individual enantiomers as chemoprotective agents. The predominant native (*R*) enantiomer is active, whereas the (*S*) antipode is inactive or has little or no biological activity. Here we provide an enantioselective high-performance liquid chromatography (HPLC) protocol for the direct and complete resolution of sulforaphane and its chiral natural homologs with different aliphatic chain lengths between the sulfinyl sulfur and isothiocyanate group, namely iberin, alyssin, and hesperin. The chromatographic separations were carried out on the immobilized-type CHIRALPAK IH-3 chiral stationary phase with amylose tris-[(*S*)-methylbenzylcarbamate] as a chiral selector. The effects of different mobile phases consisting of pure alcoholic solvents and hydroalcoholic mixtures on enantiomer retention and enantioselectivity were carefully investigated. Simple and environmentally friendly enantioselective conditions for the resolution of all chiral ITCs were found. In particular, pure ethanol and highly aqueous mobile phases gave excellent enantioseparations. The retention factors of the enantiomers were recorded as the water content in the aqueous-organic modifier (methanol, ethanol, or acetonitrile) mobile phases progressively varied. U-shaped retention maps were generated, indicating a dual and competitive hydrophilic interaction liquid chromatography (HILIC) and reversed-phase liquid chromatography retention mechanism on the CHIRALPAK IH-3 chiral stationary phase. Finally, experimental chiroptical studies performed in ethanol solution showed that the (*R*) enantiomers were eluted before the (*S*) counterpart under all eluent conditions investigated.

## 1. Introduction

**Figure 1:**
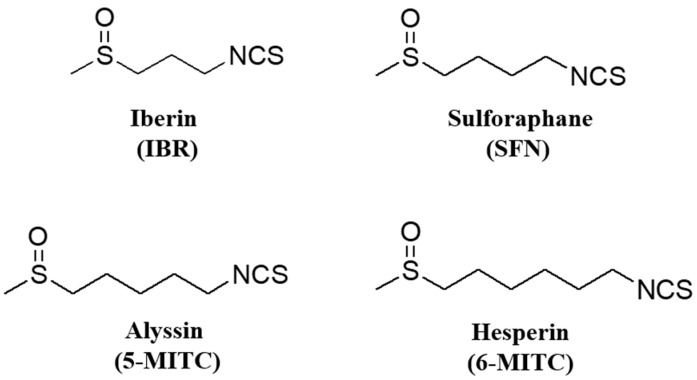
Structure of iberin (IBR), sulforaphane (SFN), alyssin (5-MITC) and hesperin (6-MITC).

A diet rich in broccoli and other cruciferous vegetables, such as cauliflower, cabbage, watercress, and Brussels sprouts, is associated with beneficial health effects, according to epidemiological and clinical evidence [1,2]. In particular, a reduced risk of developing common cancers, such as lung, colon, and breast cancer, has been observed with the consumption of such vegetables. The chemoprotective effect has been attributed to the presence of high levels of isothiocyanates (ITCs) [3,4,5,6]. Natural ITCs are not the result of plant production as such but rather, they are the result of the enzymatic activation of thioglucoside conjugates. During harvesting, chopping, and chewing of cruciferous vegetables, myrosinase is released from a separate cellular compartment. This allows glucosinolates to come into contact with the enzyme myrosinase and be hydrolyzed to ITCs. The ITCs sulforaphane (SFN) and iberin (IBR) are two of the most abundant and potent chemopreventive phytochemicals (Figure 1).

As chemopreventive agents, SFN and IBR act at both the initiation and post-initiation stages of cancer. In the first case, they act by inducing phase II detoxification enzymes such as quinone reductase and glutathione S-transferase, which are responsible for protecting DNA from reactive genotoxic metabolites, while in the second case they act by inducing apoptosis in cancer cells [7,8,9,10,11]. It is worth noting that the inductive activity of SFN is strongly influenced by the chirality of the stereogenic sulfinyl sulfur atom [12,13].

The racemization barrier of dialkylsulfinyl compounds is typically very high [14]. Therefore, the stereogenic sulfinyl sulfur atom of the side chain of SFN and IBR is configurationally stable. This allows the enantiomers to be separated even at ambient temperature [15,16].

Despite the abundant evidence for the anticancer properties of superior SFN and IBR homologues such as alyssin (5-MITC) and hesperin (6-MITC), whose structures are shown in Figure 1, nothing is known about the biological activity of individual enantiomers. 5-MITC, present in *Alyssum* plants, induces apoptosis in leukemia cells [17] and has demonstrated more potent inducer activity in hepatocellular carcinoma cell HepG2 death compared to sulforaphane [18]. Drug–drug interaction studies have showed that 5-MITC induced enhanced the anticancer activity of 5-fluorouracil in colon cancer cell lines [19]. 

6-MITC is found in the plant *Wasabi japonica* and has been shown to inhibit cell proliferation in U937 cells by inducing apoptotic cell death [20] and cell viability in human pancreatic cancer cell lines PANC-1 and BxPC-3 [21]. The pulmonary antimetastatic effect of 6-MITC was demonstrated by a reliable method for the detection of the human c-Ha-ras gene [22]. 6-MITC also induces apoptosis of human colorectal cancer cells through the p53-independent mitochondrial dysfunction pathway [23].

The enantiomeric content of 5-MITC and 6-MITC in different parts of the plant (i.e., flowers, leaves, or stems) is unknown. Such studies require the development of effective enantioseparation protocols, which are not currently available in the literature. The most widely used technique for the analysis of chiral natural and synthetic compounds is the chiral stationary phase (CSP)-based enantioselective HPLC [24]. In recent decades, considerable efforts have been made to extend the range of enantioselectivity of chromatographic methods. New chiral chromatographic supports have been commercialized and their application has allowed the chromatographic resolution of a wide range of chiral compounds from the nanoscale to the preparative scale [25,26]. In particular, amylose- and cellulose-based polysaccharide CSPs have demonstrated broad applicability and high loading capacity [27,28,29]. They have the advantage of easy derivatization to phenylcarbamate or benzoate derivatives where the phenyl rings are substituted with different groups or atoms (i.e., methyl and/or chlorine) to modulate their chiral recognition ability [30]. Polysaccharide-based CSP columns also have the potential to operate under multimodal conditions, allowing the chiral resolution of a variety of racemic compounds using either a normal phase or an aqueous eluent. However, the search for effective enantioselective conditions has not been accompanied by an intensive effort to minimize the environmental impact associated with the use of mobile phases. High solvent consumption is a serious environmental problem, especially in cases where toxic organic solvents have various effects on human health and the environment [31].

Several ways to make HPLC enantioseparation a more sustainable process have been described in the literature [32,33,34,35,36,37], including: (i) reducing the particle diameter of the chiral stationary phase or the dimensions of the HPLC column; (ii) replacing the toxic solvents commonly used for normal-phase and reversed-phase liquid chromatography, such as acetonitrile and n-hexane, with environmentally friendly and bio-based solvents. In a previous study, we reported the HPLC enantioseparation of SFN and its lower homolog IBR on immobilized-type amylose-based CSPs under multimodal eluent conditions [15].

The present study aimed to thoroughly investigate the retention and enantioselective properties of the immobilized-type CHIRALPAK IH-3 CSP towards the ITCs IBR, SFN, 5-MITC, and 6-MITC under green eluent conditions. The effect of water and biomass-accessible chemicals such as ethanol on the chromatographic resolution was evaluated in this study. To complete the work, the assignment of the absolute configuration to the individual enantiomers of the ITCs was carried out by evaluating their chiroptical properties.

## 2. Results

### 2.1. Enantioseparations with Ethanol-Based Eluents

Due to its unique properties, ethanol is classified as a green solvent. For example, it is readily available, readily biodegradable, readily recyclable, derived from renewable intermediates and feedstocks, and has acceptable and appropriate toxicity and ecotoxicity profiles [33]. Ethanol is routinely used in enantioselective HPLC in normal-phase mode mixed with n-hexane and is rarely used in reversed-phase conditions due to its high viscosity. In this study, the capability of pure ethanol to ensure the separation of the enantiomers of the natural ITC homologues IBR, SFN, 5-MITC, and 6-MITC was evaluated using the 250 mm × 4.6 mm CHIRALPAK IH-3 column containing the amylose tris-[(*S*)-methylbenzylcarbamate] as chiral selector [38,39] immobilized on 3-μm silica particles. The chromatographic data obtained at a temperature of 25 °C and a flow rate of 0.5 mL/min are shown in Table 1.

Comparing the retention and enantioseparation factor values of the analyzed ITCs, it can be observed that: (i) all compounds were baseline resolved with enantioseparation and resolution factors between 1.32 and 1.46, and 3.43 and 6.49, respectively; (ii) the best enantioselectivity was obtained for SFN (Table 1, entry 3) and the worst for 6-MITC (Table 1, entry 7); (iii) enantioseparations were obtained in less than 20 min; (iv) in all cases the (*R*) enantiomer was eluted before the (*S*) enantiomer.

To further explore the chiral resolution capability of the CHIRALPAK IH-3 CSP with ethanol, two additional types of mobile phase were used, the first obtained by adding n-hexane and the second by adding water to alcohol. As can be seen from the chromatographic results shown in Table 1, passing from pure ethanol to n-hexane-ethanol 60:40 (*v*/*v*) the enantioselectivity decreased from 1.43 to 1.23 (Table 1, entries 1 and 2) for IBR, and from 1.66 to 1.57 (Table 1, entries 3 and 4) for SFN. An opposite trend was observed for 5-MITC and 6-MITC (entries 5–8). Although the presence of n-hexane leads to better resolution for SFN, 5-MITC, and 6-IMTC, mainly due to improved retention, it seems inappropriate to use a toxic solvent such as n-hexane when complete resolution can be achieved with ethanol. 

**Figure 2:**
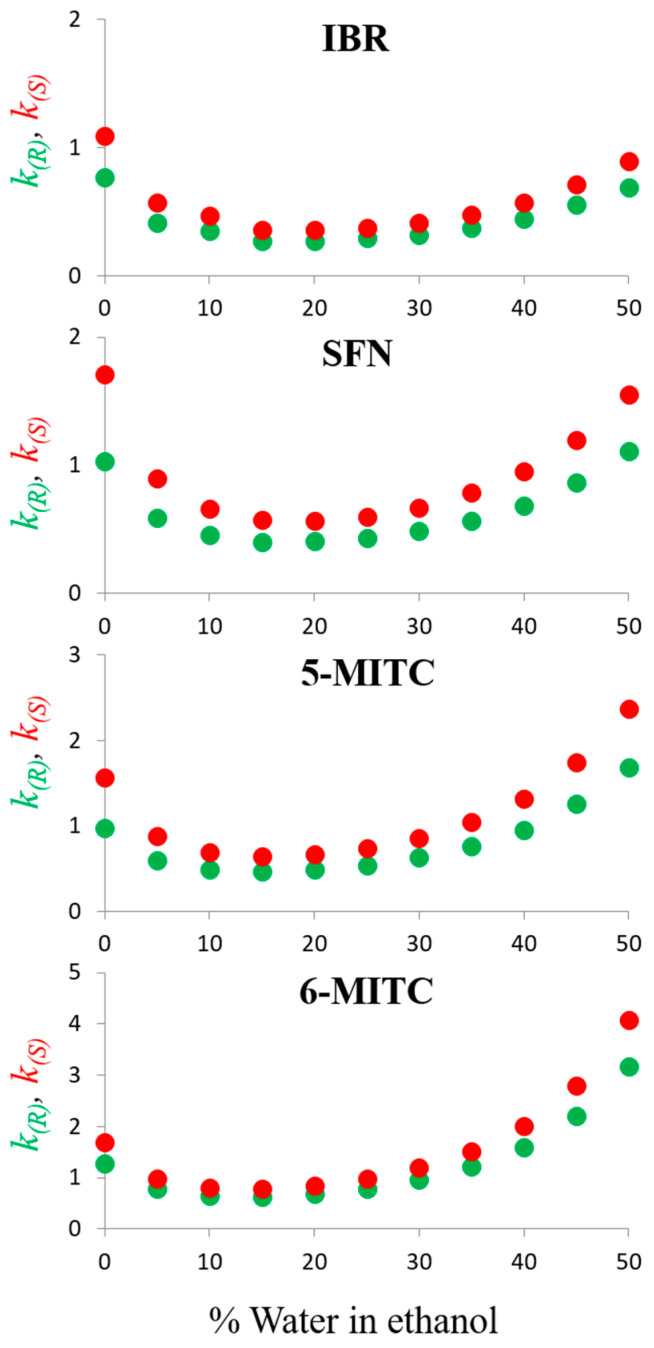
Plots of the retention factors (*k*_1_ and *k*_2_) of the enantiomers of iberin (IBR), sulforaphane (SFN), alyssin (5-MITC), and hesperin (6-MITC) as a function of the water content in the ethanol-aqueous mode. Chromatographic conditions: column, CHIRALPAK IH-3 (250 mm × 4.6 mm, 3 μm); temperature, 25 °C; flow rate, 0.5 mL/min; detection, UV at 240 nm.

Figure 2 shows the graphs obtained by plotting retention factors of the enantiomers against increasing water content from 0–50% in the binary ethanol mobile phase.

**Figure 3:**
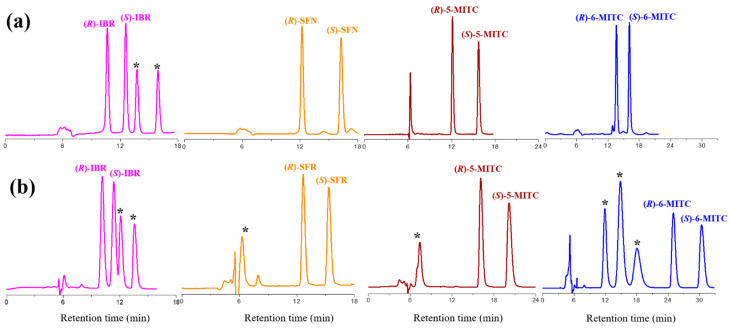
Typical chromatograms of iberin (IBR), sulforaphane (SFN), alyssin (5-MITC), and hesperin (6-MITC) obtained with ethanol (**a**) and the mixture ethanol-water 50:50 (**b**) as mobile phases. Chromatographic conditions: column, CHIRALPAK IH-3 (250 mm × 4.6 mm, 3 μm); temperature, 25 °C; flow rate, 0.5 mL/min; detection, UV at 240 nm. *: impurities.

It is interesting to note that in the retention maps, a crossover water level (i.e., 15%) defines two distinct retention ranges. In the first, from 0% to 15% water, the retention of the enantiomers decreases slightly, whereas from 15% to 50% it increases progressively. Thus, before the crossover point, water acts as a typical normal phase modifier, weakening the polar interactions established between fragments of ITC and polar active sites of the polysaccharide-type selector. This trend is typical of an HILIC retentive mechanism [40,41,42,43,44,45,46]. Beyond the critical mobile phase composition, the increase in water in the mobile phase triggers an increase in retention typical of the reversed-phase (RP) mechanism. The longer the length of the aliphatic ITC chain, the more pronounced the slope of the RP branch of the retention maps. A comparison of the chromatograms obtained by resolving four ITCs using ethanol and a 50:50 (*v*/*v*) ethanol/water mixture as mobile phases is shown in Figure 3.

Pure ethanol gave better resolution in all cases, and for the more hydrophobic 5-MITC and 6-MITC the retention times recorded under RP conditions were significantly higher than those recorded with pure alcohol (see Figure S1 in the Supplementary Materials). From Figure 3 it can be seen that the unknown impurities present in the commercial samples of 5-MITC and 6-MITC (marked with an asterisk) were eluted earlier than enantiomeric peaks in the RP mode, whereas they were retained to a greater extent by ethanol alone. It is therefore possible to avoid the interference of such polar impurities in the analysis of ITCs with the correct choice of mobile phase. As shown in previous work [15], the additional peaks in the IBR chromatogram belong to the enantiomers of the chiral thiocarbamate ester derivative formed during storage of the commercial IBR sample in ethanol solution.

### 2.2. Enantioseparations with Methanol-Based Eluents

Compared to ethanol, methanol cannot be considered a fully green solvent due to its higher toxicity. However, given its good biodegradability and low disposal costs, methanol can be classified as an environmentally friendly solvent [33]. In this context, the enantioseparation of ITCs on the CHIRALPAK IH-3 column was evaluated in pure methanol and with methanol–water mixtures containing 5%, 10%, 15%, 20%, 25%, 30%, 35%, and 40% of water. The retention, enantioseparation, and resolution factors obtained are shown in the plots of *k*_1,2_/*α*/*Rs* vs. %water in Figure S2. As shown in Figure S2, the trend of the enantiomer retention as a function of increasing water content in methanol is consistent with a prevailing retention mechanism, i.e., the analytes interact preferentially with the stationary phase through hydrophobic interactions rather than hydrophilic interactions. The retentive mechanism reflects the ability of methanol to form a water-like shielding layer on the hydrophilic surface of the stationary phase through strong hydrogen bonds. Thus, the presence of methanol abolishes the HILIC domain of the retention maps, allowing hydrophobic interactions to drive the retention process. Replacing ethanol with methanol, either as the sole component of the mobile phase or as an alcohol modifier mixed with water, had the effect of reducing the enantioselectivity and resolution of the four ITCs investigated. For example, the resolution factor values were 3.48 and 3.80 for SFN and 5-MITC with methanol and 6.49 and 5.03 with ethanol (for details see the chromatographic data with pure methanol and the mixture methanol:water 70:30 (*v*/*v*) given in Table S1). Therefore, the use of methanol instead of ethanol does not improve either the performance of the enantioselective analysis or the sustainability of the method.

### 2.3. Enantioseparations with Acetonitrile–Water Eluents

Despite the remarkable selective properties demonstrated as an organic modifier in HILIC and RP modes, acetonitrile is classified as a hazardous solvent due to its inherent toxicity and the stringent requirements for its disposal [47]. 

The strategy of switching from the use of acetonitrile to more environmentally friendly elution conditions is desirable and should be encouraged [48]. Another option for a greener analysis is the use of acetonitrile in a mixture with a high concentration of water. This is the case when the column exhibits the dualistic HILIC/RP behavior. Under ACN conditions, the curvature of the U-shaped retention maps is significantly more pronounced than that obtained with ethanol. This enables short analysis times to be achieved with a high water content in the mobile phase.

**Figure 4:**
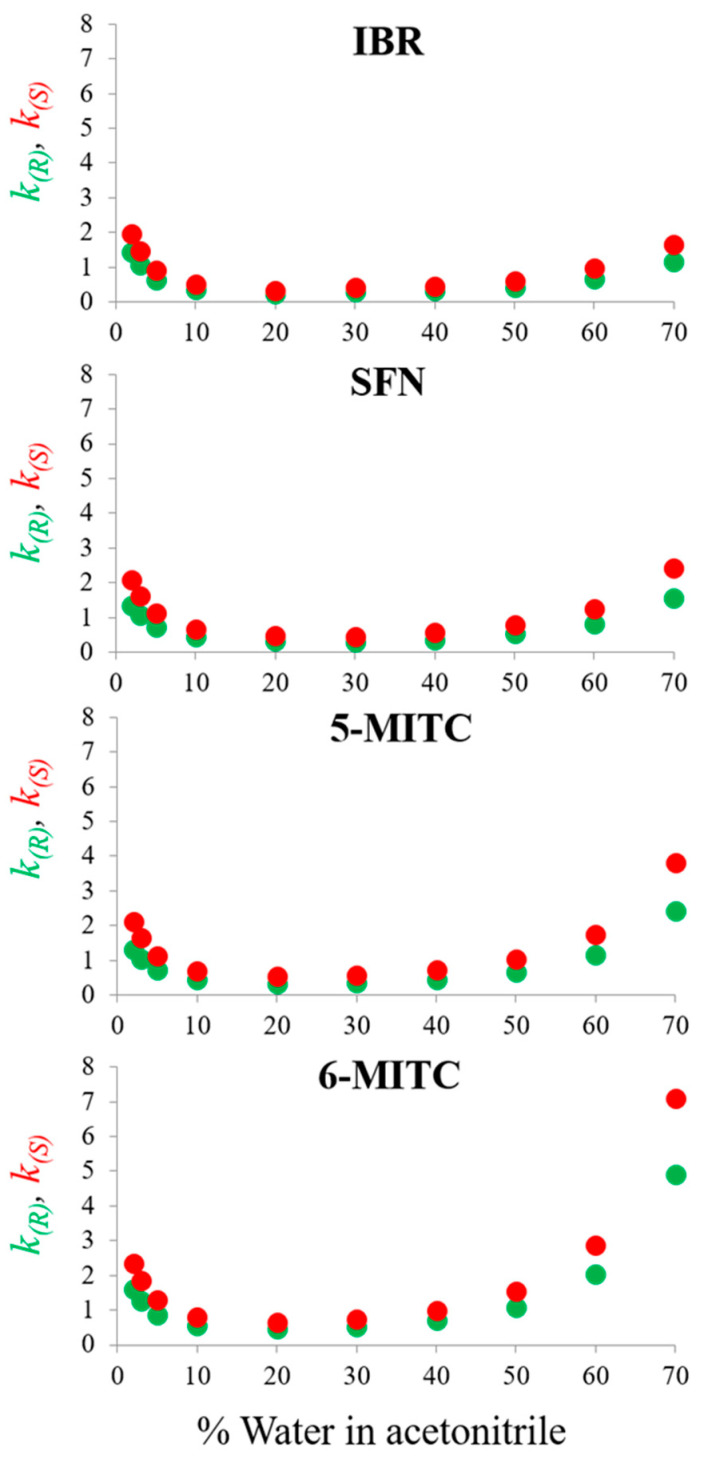
Plots of the retention factors (*k*_1_ and *k*_2_) of the enantiomers of iberin (IBR), sulforaphane (SFN), alyssin (5-MITC), and hesperin (6-MITC) as a function of the water content in the acetonitrile-aqueous mode. Chromatographic conditions: column, CHIRALPAK IH-3 (250 mm × 4.6 mm, 3 μm); temperature, 25 °C; flow rate, 0.5 mL/min; detection, UV at 240 nm.

The trend of the retention plots obtained from the analysis of the four ITCs with acetonitrile–water binary mixtures confirms this expectation. As can be seen in Figure 4, the retention times of enantiomers of IBR and SFN with 2% of water in acetonitrile are very similar to those pertinent to 70% of water.

**Figure 5:**
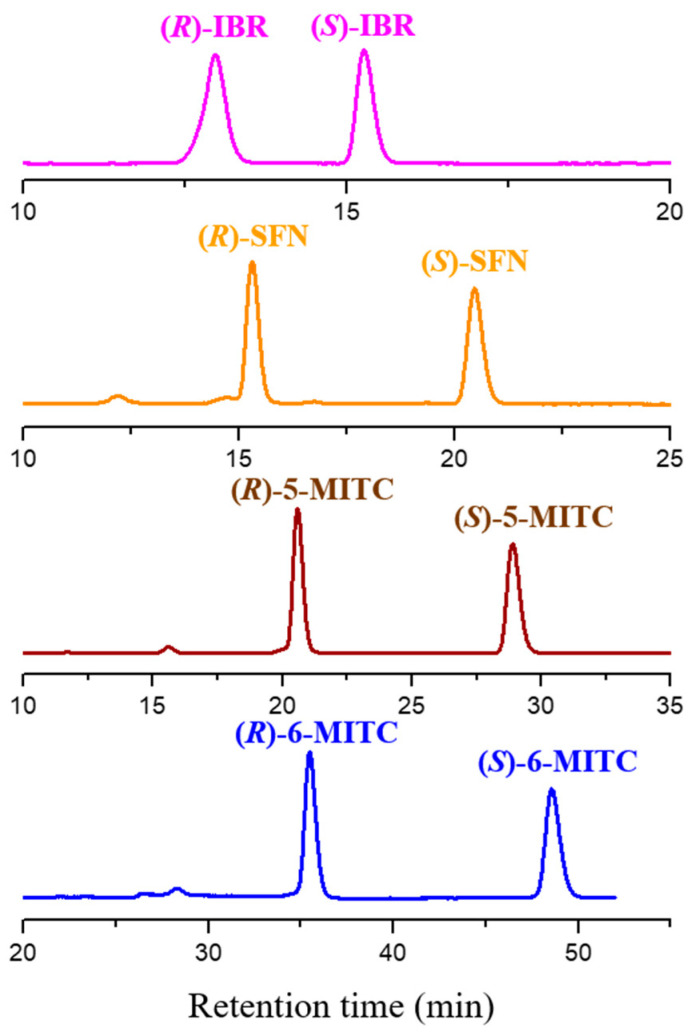
Typical chromatograms of iberin (IBR), sulforaphane (SFN), alyssin (5-MITC), and hesperin (6-MITC) obtained with the mixture acetonitrile–water 30:70 as a mobile phase. Chromatographic conditions: column, CHIRALPAK IH-3 (250 mm × 4.6 mm, 3 μm); temperature, 25 °C; flow rate, 0.5 mL/min; detection, UV at 240 nm.

Superior homologs of IBR and SFN, namely 5-MITC and 6-MITC, which have a longer aliphatic chain between the stereogenic sulfur and isothiocyanate groups, showed stronger retention with acetonitrile–water 30:70 (*v*/*v*). Analysis of the chromatograms shown in Figure 5 indicates that the elution of 5-MITC was completed within 30 min and that of 6-MITC within 50 min, with acetonitrile consumption of 4.5 and 7.5 mL, respectively.

The mobile phase consumption could be reduced by scaling down the analytical conditions using a miniaturized column packed with the same chiral chromatographic support (i.e., a column with a geometry of 100 mm × 4.6 mm, which is commercially available but which we do not have in stock). This approach could allow the flow rate to be increased from 0.5 mL/min to 1.0 mL/min, which would also reduce the analysis time. The use of a column with reduced geometry should not compromise the completeness of the separation, as a high level of resolution was obtained with the acetonitrile–water 30:70 (*v*/*v*) mobile phase (Figure 5). Under these conditions, the resolution factor values for IBR, SFN, 5-MITC, and 6-MITC were 4.21, 8.79, 10.27, and 10.35, respectively. Additional chromatographic data are reported in Table S2. The enantioselectivity values were essentially unaffected by the addition of water to acetonitrile. The improvement in resolution was therefore mainly due to an increase in retention. 

As a final remark of this work, it is interesting to highlight the inversion of the enantiomeric elution order of IBR and SFN on the CHIRALPAK IH-3 CSP (i.e., (*R*) enantiomer eluted before the (*S*) antipode) in all the elution conditions studied with respect to that observed for the immobilized-type CHIRALPAK IA-3, CHIRALPAK ID-3, CHIRALPAK IE-3, CHIRALPAK IF-3, and CHIRALPAK IG-3 CSPs and the coated-type CHIRALPAK AD CSP [15]. The same sense of chiral recognition operates on CHIRALPAK IH-3 CSP for the superior homologs 5-MITC and 6-MITC, with the (*R*) enantiomer retained less than the (*S*) counterpart. All the CSPs mentioned above share: (i) the amylose backbone; (ii) the hydrogen bonding functional groups C=O and NH; and (iii) an aromatic group of the polymer side chains [29,30,38,39]. The amylose tris-[(*S*)-methylbenzylcarbamate] selector is characterized by the presence of an additional stereogenic center with (*S*) absolute configuration located on the phenyl-1-ethyl group. This structural feature appears to be crucial for enantioselective interactions with the ITCs studied. 

## 3. Materials and Methods

### 3.1. Materials

IBR, SFN, 5-MITC, and 6-MITC were purchased from Cayman Chemical (Ann Arbor, MI, USA), Vinci-Biochem (Vinci, Italy), AmBeed (Arlington Heights, IL, USA), and Alpha Chemistry (New York, NY, USA), respectively. HPLC-grade solvents (n-hexane, ethanol, acetonitrile, methanol, and water) were purchased from Sigma-Aldrich (Milan, Italy). 

HPLC enantioseparations were performed by using the commercially available stainless-steel CHIRALPAK^®^ IH-3 (250 mm × 4.6 mm, 3 μm) column (Chiral Technologies Europe, Illkirch, France).

### 3.2. Instruments and Chromatographic Conditions

The enantiomeric separations of IBR, SFN, 5-MITC, and 6-MITC were performed on a Jasco LC-4000 UHPLC (Jasco, Tokyo, Japan). This instrument included a binary pump system with a maximum flow rate of 2 mL min^−1^, an autosampler with an injection loop volume of 50 μL (used in partial loop mode), an MD-4010 photodiode array detector with a 16 μL internal flow cell, and a column oven. Data acquisition, data processing, and instrument control were performed using Jasco ChomNAV software.

Fresh standard solutions of ITCs for HPLC analysis were prepared by dissolving the analytes in dichloromethane or ethanol. Injection volumes were 10–30 μL.

ECD spectra were measured in a 0.1 cm path length quartz cell at 25 °C using a Jasco model J-700 spectropolarimeter. The spectra are averaged over four instrumental scans and the intensities are expressed as ellipticity values (mdeg).

### 3.3. Absolute Configuration Assignment

The enantiomers of IBR and SFN of known stereochemistry were used to determine their enantiomeric elution order on the CHIRALPAK IH-3 CSP [15]. In order to determine the enantiomeric elution order of 5-MITC and 6-MITC on the CHIRALPAK IH-3 CSP, enantiopure forms (ee > 99%) of the two ITCs were isolated by multiple enantioseparations on the 250 mm × 4.6 mm CHIRALPAK IH-3 column using ethanol as mobile phase. The collected enantiomers were then subjected to electronic circular dichroism (ECD) analysis. The ECD spectra of the first eluted enantiomers of 5-MITC and 6-MITC on the CHIRALPAK IH-3 CSP are shown in Figure S3. A single negative ECD band was observed in the 300 to 198 nm spectral region, at around 200 nm, which is diagnostic for the (*R*) absolute configuration according to the ECD properties of the lower homologues IBR and SFN [15].

## 4. Conclusions

The increasing cost of disposal and recycling of toxic organic solvents is driving industry and academia towards an environmentally oriented approach to the development of analytical methods for testing the purity of pharmaceuticals and bioactive compounds.

In an effort to find new and innovative methods of conducting enantioselective HPLC, the chiral resolution capacity of the amylose-based immobilized-type CHIRALPAK IH-3 CSP towards the four chemopreventive homologs IBR, SFN, 5-MITC, and 6-MITC was investigated by selecting green eluent conditions. Pure ethanol and the highly aqueous mixture acetonitrile–water 30:70 were found to provide good enantioselective and sustainable conditions. The systematic evaluation of the chromatographic data by progressively varying the composition of the aqueous eluents confirms the findings of previous works on the dual HILIC/RP retention behavior of polysaccharide-based CSPs and highlights the applicability of this type of chiral chromatographic support for chiral analysis in the underexplored field of sustainable HPLC.

For the first time, the enantiomers of 5-MITC and 6-MITC have been separated by direct enantioselective HPLC. This makes it possible (i) to study the pharmacodynamic and pharmacokinetic properties of the individual enantiomers; (ii) to check the enantiomeric composition of natural samples; and (iii) to monitor more easily the stereochemical course of the asymmetric synthesis used for their preparation. On this last point, it is necessary to mention that control of the enantiomeric excess and assignment of the absolute configuration of the enantiomers of ITCs extracted from a natural matrix [49] or produced by asymmetric synthesis [50] are usually carried out by means of polarimetric analysis. The determination of the specific rotation is carried out in a solution of chloroform, a highly toxic solvent. The EPA (Environmental Protection Agency) classifies chloroform as a Group B2 substance, likely to be carcinogenic to humans. Furthermore, chiral and achiral impurities that may be present in the reaction product can also interfere with polarimetric analysis. Since ITCs are oils at room temperature and therefore cannot be subjected to X-ray diffraction analysis, ECD analysis of ethanol solutions of individual enantiomers appears to be the more appropriate and environmentally friendly method for determining their absolute configuration. Finally, it is worth noting that research into natural bioactive compounds for the prevention and treatment of cancer is increasing. Therefore, we believe that the results of the present work may also be useful in the evaluation of single enantiomer ITCs as potential adjuvant antitumor agents.

## Figures and Tables

**Table 1:** Retention factor (*k*_1_) for the first eluting enantiomer, enantioseparation (*α*), and resolution (*Rs*) factors of iberin (IBR), sulforaphane (SFN), alyssin (5-MITC), hesperin (6-MITC) in ethanol-based conditions. Chromatographic conditions: column, CHIRALPAK IH-3 (250 mm × 4.6 mm, 3 μm); temperature, 25 °C; flow rate, 1 mL/min; detection, UV at 240 nm.

Entry	Compound	Mobile Phase	*k*_1_	*α*	*Rs*
1	IBR	Ethanol	0.73	1.43	3.65
2		n-Hexane/Ethanol 60:40	2.78	1.23	3.31
3	SFN	Ethanol	0.98	1.66	6.49
4		n-Hexane/Ethanol 60:40	3.32	1.57	8.85
5	5-MITC	Ethanol	0.93	1.61	5.03
6		n-Hexane/Ethanol 60:40	2.97	1.64	8.75
7	6-MITC	Ethanol	1.22	1.32	3.43
8		n-Hexane/Ethanol 60:40	3.58	1.36	6.27

## Data Availability

The data presented in this study are available in article and Supplementary Materials.

## Supplementary Materials

The following supporting information can be downloaded at: https://www.mdpi.com/article/10.3390/molecules29122895/s1, Table S1. Retention factor (*k*_1_) for the first eluting enantiomer, enantioseparation (*α*) and resolution (*Rs*) factors of iberin (IBR), sulforaphane (SFN), alyssin (5-MITC), hesperin (6-MITC) in methanol-based conditions. Table S2. Retention factor (*k*_1_) for the first eluting enantiomer, enantioseparation (*α*) and resolution (*Rs*) factors of iberin (IBR), sulforaphane (SFN), alyssin (5-MITC), hesperin (6-MITC) using acetonitrile-water 30:70 (*v*/*v*) as a mobile phase. Figure S1. Plots of the enantioseparation and resolution factors of iberin (IBR), sulforaphane (SFN), alyssin (5-MITC) and hesperin (6-MITC) as a function of the water content in the ethanol-aqueous mode. Chromatographic conditions: column, CHIRALPAK IH-3 (250 × 4.6 mm, 3 µm); temperature, 25 °C; flow rate, 0.5 mL/min; detection, UV at 240 nm. Figure S2. Plots of the retention, enantioseparation and resolution factors of iberin (IBR), sulforaphane (SFN), alyssin (5-MITC) and hesperin (6-MITC) as a function of the water content in the methanol-aqueous mode. Chromatographic conditions: column, CHIRALPAK IH-3 (250 × 4.6 mm, 3 µm); temperature, 25 °C; flow rate, 0.5 mL/min; detection, UV at 240 nm. Figure S3. CD spectra of the first eluted enantiomer of 5-MITC (wine line) and 6-MITC (blue line) on the CHIRALPAK IH-3 CSP recorded in ethanol at 25 °C.
