# Supplementary material for: Green HPLC Enantioseparation of Chemopreventive Chiral Isothiocyanates Homologs on an Immobilized Chiral Stationary Phase Based on Amylose tris-[(S)-α-Methylbenzylcarbamate]
Source: Molecules. 2024 Jun 18;29(12):2895. doi: 10.3390/molecules29122895 (PMC11206679; doi:10.3390/molecules29122895)

Supplementary Materials

Green HPLC Enantioseparation of Chemopreventive Chiral Isothiocyanates Homologs on an Immobilized Chiral Stationary Phase Based on Amylose tris-[(*S*)-α-Methylbenzylcarbamate]

Francesca Romana Mammone [1], Alessia Panusa [1], Roberta Risoluti [2] and Roberto Cirilli [1,*]

[1] National Centre for the Control and Evaluation of Medicines, Chemical Medicines Unit, Istituto Superiore di Sanità, Viale Regina Elena 299, 00161 Rome, Italy; francescaromana.mammone@uniroma1.it (F.R.M.); alessia.panusa@iss.it (A.P.)

[2] Department of Chemistry, "Sapienza" University of Rome, P.le A. Moro 5, 00185 Rome, Italy; roberta.risoluti@uniroma1.it

* Correspondence: roberto.cirilli@iss.it

Table S1. Retention factor (k_1) for the first eluting enantiomer, enantioseparation (α) and resolution (Rs) factors of iberin (IBR), sulforaphane (SFN), alyssin (5-MITC), hesperin (6-MITC) in methanol-based conditions.

Compound	Mobile phase	k_1	a	Rs
IBR	Methanol	0.37	1.30	2.76
	Methanol/water 70:30	0.61	1.25	1.85
SFN	Methanol	0.47	1.35	3.48
	Methanol/water 70:30	0.85	1.31	3.34
5-MITC	Methanol	0.48	1.35	3.80
	Methanol/water 70:30	1.14	1.31	3.59
6-MITC	Methanol	0.59	1.22	2.78
	Methanol/water 70:30	1.18	1.26	3.62

Chromatographic conditions: column, CHIRALPAK IH-3 (250 x 4.6 mm, 3 μm); temperature, 25 °C; flow rate, 0.5 mL/min; detection, UV at 240 nm.

Table S2. Retention factor (k_1) for the first eluting enantiomer, enantioseparation (α) and resolution (Rs) factors of iberin (IBR), sulforaphane (SFN), alyssin (5-MITC), hesperin (6-MITC) using acetonitrile-water 30:70 (v/v) as a mobile phase.

Compound	k_1	a	Rs
IBR	1.16	1.42	4.21
SFN	1.55	1.56	8.79
5-MITC	2.43	1.57	10.27
6-MITC	4.91	1.44	10.35

Chromatographic conditions: column, CHIRALPAK IH-3 (250 x 4.6 mm, 3 μm); temperature, 25 °C; flow rate, 0.5 mL/min; detection, UV at 240 nm.

Figure S1. Plots of the enantioseparation and resolution factors of iberin (IBR), sulforaphane (SFN), alyssin (5-MITC) and hesperin (6-MITC) as a function of the water content in the ethanol-aqueous mode. Chromatographic conditions: column, CHIRALPAK IH-3 (250 x 4.6 mm, 3 μm); temperature, 25 °C; flow rate, 0.5 mL/min; detection, UV at 240 nm.

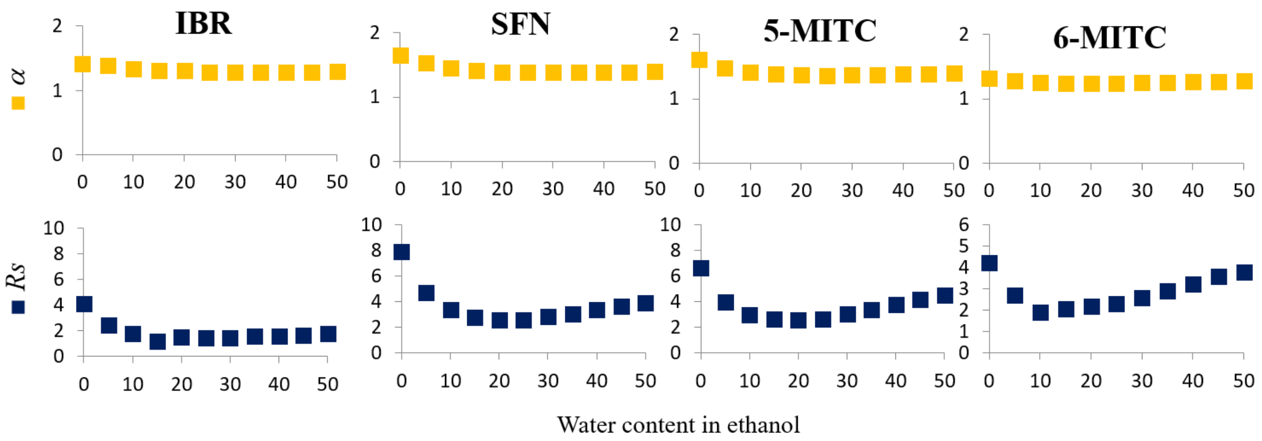

Figure S2. Plots of the retention, enantioseparation and resolution factors of iberin (IBR), sulforaphane (SFN), alyssin (5-MITC) and hesperin (6-MITC) as a function of the water content in the methanol-aqueous mode. Chromatographic conditions: column, CHIRALPAK IH-3 (250 x 4.6 mm, 3 μm); temperature, 25 °C; flow rate, 0.5 mL/min; detection, UV at 240 nm.

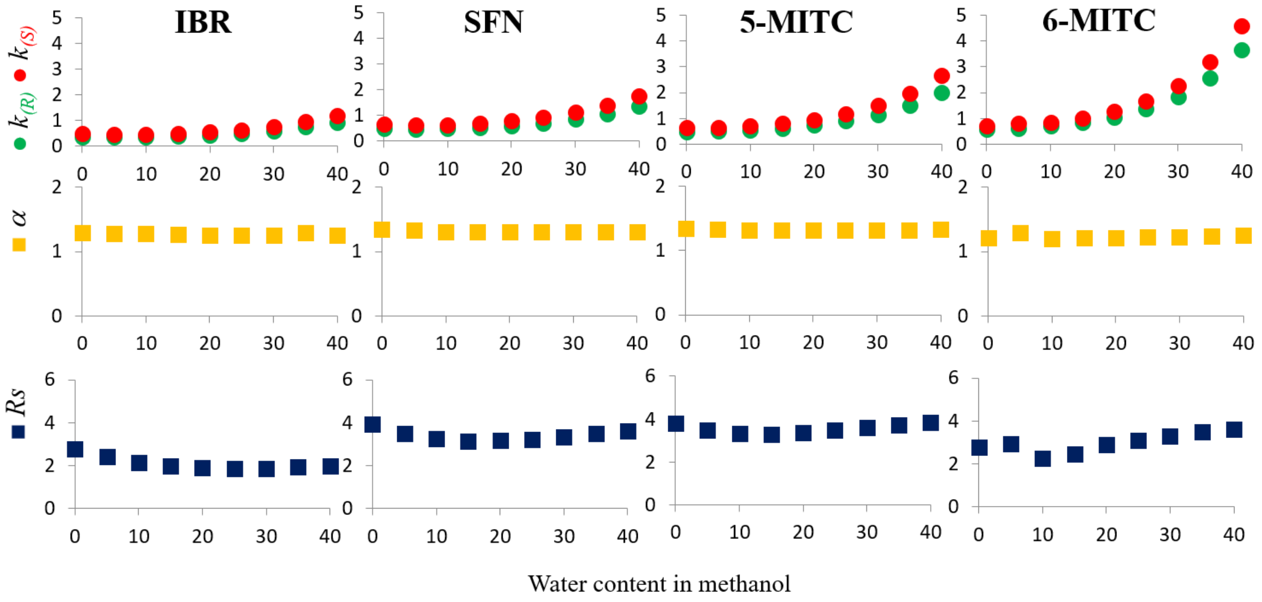

Figure S3. CD spectra of the first eluted enantiomer of 5-MITC (wine line) and 6-MITC (blue line) on the CHIRALPAK IH-3 CSP recorded in ethanol at 25 °C.

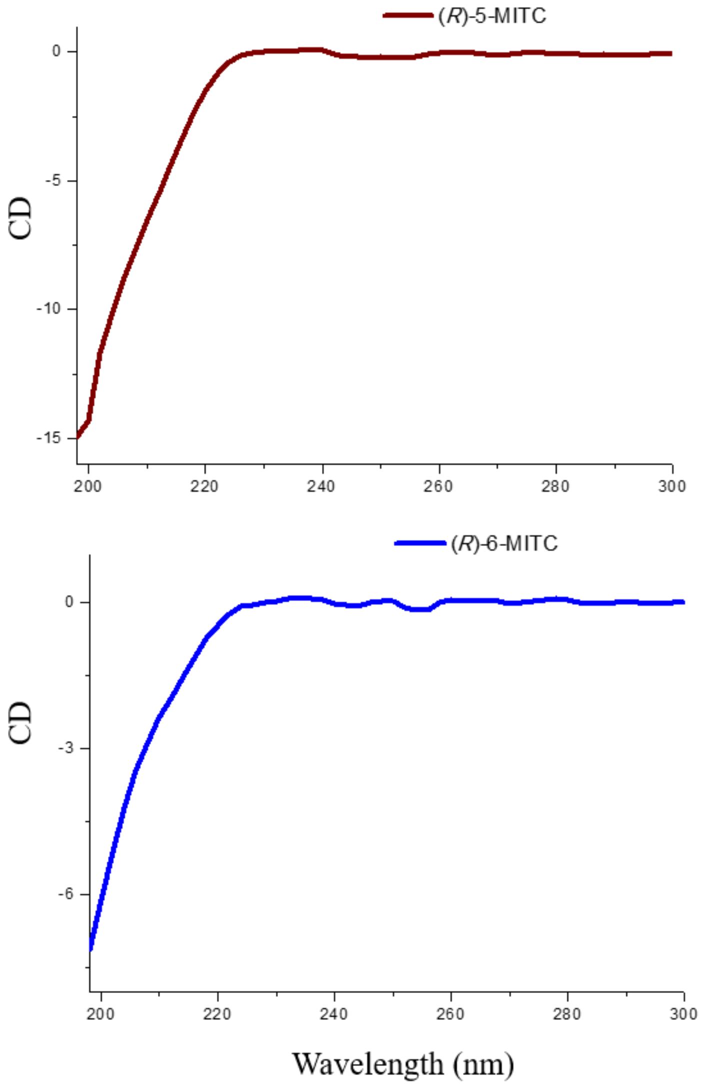

Supplement: Supplementary file 1 [file molecules-29-02895-s001.zip › molecules-3019072-supplementary.pdf]
